# CDK7 Inhibitor THZ1 Induces the Cell Apoptosis of B-Cell Acute Lymphocytic Leukemia by Perturbing Cellular Metabolism

**DOI:** 10.3389/fonc.2021.663360

**Published:** 2021-04-06

**Authors:** Tuersunayi Abudureheman, Jing Xia, Ming-Hao Li, Hang Zhou, Wei-Wei Zheng, Neng Zhou, Rong-Yi Shi, Jian-Min Zhu, Li-Ting Yang, Li Chen, Liang Zheng, Kai Xue, Kai Qing, Cai-Wen Duan

**Affiliations:** ^1^Key Laboratory of Pediatric Hematology and Oncology Ministry of Health and Pediatric Translational Medicine Institute, Shanghai Children's Medical Center, Shanghai Jiao Tong University School of Medicine, Shanghai, China; ^2^Department of Pathology, The Affiliated Hospital of Youjiang Medical University for Nationalities, Baise, China; ^3^Shanghai Blood Center, Shanghai, China; ^4^Department of Pharmacology and Chemical Biology, Shanghai Collaborative Innovation Center for Translational Medicine, Shanghai Jiao Tong University School of Medicine, Shanghai, China; ^5^Department of Hematology, Institute of Hematology, Changhai Hospital Affiliated to Navy Military Medical University, Shanghai, China; ^6^State Key Laboratory of Medical Genomics, National Research Center for Translational Medicine at Shanghai, Shanghai Institute of Hematology, Ruijin Hospital Affiliated to Shanghai Jiao Tong University School of Medicine, Shanghai, China

**Keywords:** B-cell acute lymphocytic leukemia, CDK7 inhibitor, cell apoptosis, metabolism, c-MYC

## Abstract

B-cell acute lymphocytic leukemia (B-ALL) is a malignant blood cancer that develops in children and adults and leads to high mortality. THZ1, a covalent cyclin-dependent kinase 7 (CDK7) inhibitor, shows anti-tumor effects in various cancers by inhibiting cell proliferation and inducing apoptosis. However, whether THZ1 has an inhibitory effect on B-ALL cells and the underlying mechanism remains obscure. In this study, we showed that THZ1 arrested the cell cycle of B-ALL cells *in vitro* in a low concentration, while inducing the apoptosis of B-ALL cells *in vitro* in a high concentration by activating the apoptotic pathways. In addition, RNA-SEQ results revealed that THZ1 disrupted the cellular metabolic pathways of B-ALL cells. Moreover, THZ1 suppressed the cellular metabolism and blocked the production of cellular metabolic intermediates in B-ALL cells. Mechanistically, THZ1 inhibited the cellular metabolism of B-ALL by downregulating the expression of c-MYC-mediated metabolic enzymes. However, THZ1 treatment enhanced cell apoptosis in over-expressed c-MYC B-ALL cells, which was involved in the upregulation of p53 expression. Collectively, our data demonstrated that CDK7 inhibitor THZ1 induced the apoptosis of B-ALL cells by perturbing c-MYC-mediated cellular metabolism, thereby providing a novel treatment option for B-ALL.

## Introduction

B-cell acute lymphocytic leukemia (B-ALL) is a lymphocytic malignancy that frequently occurs in children and adults, which can infiltrate the bone marrow and extramedullary sites. With recent advances in multi-modal chemotherapy regimens, the 5-year overall survival has reached 90% in children with B-ALL, whereas the outcome of older patients (≥40 years) remains poor (1). Moreover, the refractory and relapsed B-ALL patients show no response to existing therapeutic drugs, and a proportion of patients cannot tolerate the side-effects of multi-drug combination therapy (2). Therefore, identifying new alternative drugs for the treatment of B-ALL is necessary.

Cyclin-dependent kinases (CDKs) are serine/threonine protein kinases that play pivotal regulatory roles in gene transcription (e.g., CDKs 7–13 and 19–20) and cell cycle progression (e.g., CDKs 1–6 and 14–18) (3, 4). Cyclin-dependent protein 7 (CDK7) is a member of the CDK family, which forms the trimeric complex CDK-activating kinase by combining with cyclin H and MAT1 and renders CDK7 uniquely involved in the regulation of transcription and cell cycle transitions. CDK7 regulates mRNA transcription initiation and elongation by phosphorylating Ser-2 and Ser-5 of the carboxyl terminal domain (CTD) of RNA polymerase II (RNA Pol II) (5–7). Several studies have reported that aberrant expression of CDK7 has been found in many types of tumor, such as hepatocellular carcinoma, gastric cancer, colorectal cancer, and epithelial ovarian cancer, and correlated with poor prognosis (8–10). Therefore, CDK7 is a potential drug target for the treatment of tumors. THZ1 is the first selective and covalent CDK7 inhibitor, which shows anti-tumor effects in various cancers by inhibiting cell proliferation and inducing apoptosis (11–14). However, whether THZ1 has an inhibitory effect on B-ALL cells and the underlying mechanism remains unclear.

Metabolic reprogramming is a hallmark of tumor cells. The cellular metabolism of tumor cells is reprogrammed to fulfill the excessive metabolic demands for uncontrolled cell proliferation (15, 16). Glycolysis, also known as the Warburg effect, is the initial step in glucose metabolism, which consumes a large number of glucose and results in lactate production even under adequate oxygen conditions. The metabolic intermediates of glycolysis provide the materials for nucleotide synthesis in tumor cells (17). Several studies have suggested that cell cycle progression is closely coupled to the cellular metabolism of tumors. CDK6 inhibits the catalytic activity of two key enzymes, 6-phosphofructokinase and pyruvate kinase M2, in the glycolytic pathway and promotes the cell cycle progression of tumors (18). CDK8 promotes glycolysis by regulating the expression of many components of the glycolytic cascade in tumor cells (19). Recent studies have demonstrated that CDK7 regulates the expression of proto-oncogenes, including MYC and MCL1. MYC-amplified tumors are highly vulnerable to the treatment of CDK7 inhibitors, such as THZ1 (20–22). MYC facilitates tumor progression by modulating the expression of genes that are involved in the cellular metabolism of tumor cells (23, 24). CDK7 inhibitors induce cell cycle arrest and apoptosis of lung cancer cells via blocking the glucose consumption or interfering with cancer metabolism (25, 26). However, whether CDK7 inhibitors induce cell apoptosis in B-ALL by suppressing MYC-mediated cellular metabolism remains unknown.

In this study, we demonstrated that THZ1 induced the apoptosis of B-ALL cells *in vitro* in a high concentration. Moreover, THZ1 suppressed the cellular metabolism and blocked the production of cellular metabolic intermediates in B-ALL cells. Mechanistically, THZ1 inhibited the cellular metabolism of B-ALL by downregulating the expression of c-MYC-mediated metabolic enzymes. However, THZ1 treatment enhanced the cell apoptosis in over-expressed c-MYC B-ALL cells, which was involved in the upregulation of p53 expression. Therefore, these findings provide a novel clinical treatment option for B-ALL.

## Materials and Methods

### Clinical Samples

Bone marrow samples from children with B-ALL were collected as part of the initial diagnostic investigations at Shanghai Children's Medical Center (SCMC). Sample usage and protocols were approved and supervised by the SCMC Ethics Committee. All the samples were stored at SCMC and analyzed in a blind manner. B-ALL cells were seeded at a density of 1 × 10^6^ cells/ml in a STEMSPAN medium supplemented with 20 ng/ml recombinant human IL3 (rhIL3), 10 ng/ml rhIL7, 10 ng/ml rhIL6, 10 ng/ml rhIL2, 10 ng/ml rhIGF-1, 20 ng/ml rhFlt3L, and 10 ng/ml rhVcam1. B-ALL cells were treated with or without 500 nM of THZ1 hydrochloride (Med Chem Express (MCE) for 24 h, and the apoptotic percentage was detected by flow cytometry (BD Biosciences).

### Culture of Cell Lines

Human cell lines, NALM6 and REH, were obtained from the American Type Culture Collection (ATCC) (Manassas, VA) and cultured in RPMI-1640 medium supplemented with 10% fetal bovine serum (Gibco) and 1% penicillin–streptomycin (Gibco) at 37°C in a 5% CO_2_ atmosphere. Cell lines were routinely evaluated using short tandem repeat (STR) DNA profiling, and all cell lines were negative for mycoplasma. Overexpression stable cell lines of c-MYC and vector plasmids were acquired from Dr. Li T (SCMC, China). Furthermore, overexpression efficiency was verified by Western blot analysis.

### Drug Sensitivity Assay

For drug sensitivity assay, cells (10,000 cells per well) were plated in a 96-well plate and then treated with a gradient concentration of THZ1 for 72 h. Cell viability was determined using CTG (Promega CellTiter-Glo™ Luminescent Cell Viability Assay Kit) according to the manufacturer's protocol. The absorbance optical density of 405 nm was recorded using a microplate reader (Synerge2; BioTek Instruments, Winooski, VT, USA), and the half-maximal inhibitory concentration (IC50) was calculated using GraphPad Prism (GraphPad Software, La Jolla, CA, USA).

### Real-Time PCR Analysis

Total RNA was isolated from cell lines by a TRIzol reagent (Life technologies). Then, cDNA reverse was transcribed, and real-time polymerase chain reaction was performed in accordance with the instructions of PrimeScript RT reagent kit (Vazyme). Real-time PCR was conducted using the YEASEN Hieff qPCR SYBR Green Master Mix. The relative mRNA expression was calculated by the comparative Ct method using β-actin as a control. The primers are listed as follows:

**Table:** 

**Gene list**	**Sequence (5^**′**^-3^**′**^)**
β-actin Forward Primer	TGCCGACAGGATGCAGAAG
β-actin Reverse Primer	GCCGATCCACACGGAGTACT
CDK1 Reverse Primer	TCCTGCATAAGCACATCCTGA
CDK2 Forward Primer	CCAGGAGTTACTTCTATGCCTGA
CDK2 Reverse Primer	TTCATCCAGGGGAGGTACAAC
CDK4 Forward Primer	ATGGCTGCCACTCGATATGAA
CDK4 Reverse Primer	TCCTCCATTAGGAACTCTCACAC
CDK6 Forward Primer	TCTTCATTCACACCGAGTAGTGC
CDK6 Reverse Primer	TGAGGTTAGAGCCATCTGGAAA
CDK7 Forward Primer	ATGGCTCTGGACGTGAAGTCT
CDK7 Reverse Primer	GCGACAATTTGGTTGGTGTTC
E2F1 Forward Primer	ACGCTATGAGACCTCACTGAA
E2F1 Reverse Primer	TCCTGGGTCAACCCCTCAAG
E2F2 Forward Primer	CGTCCCTGAGTTCCCAACC
E2F2 Reverse Primer	GCGAAGTGTCATACCGAGTCTT
BCL-XL Forward Primer	TCAGGCTGCTTGGGATAAAG
BCL-XL Reverse Primer	AGGCTTCTGGAGGACATTTG
BCL2 Forward Primer	AAGATTGATGGGATCGTTGC
BCL2 Reverse Primer	TGTGCTTTGCATTCTTGGAC
XIAP Forward Primer	ACCGTGCGGTGCTTTAGTT
XIAP Reverse Primer	TGCGTGGCACTATTTTCAAGATA
P21 Forward Primer	GAAGAGGCTGGTGGCTATTT
P21 Reverse Primer	TAAAGGATGACAAGCAGAGAGC
P53 Forward Primer	GTACCACCATCCACTACAACTAC
P53 Reverse Primer	CTTTGAGGTGCGTGTTTGTG

### RNA-SEQ Analysis

Total cellular RNA was isolated using the TRIzol reagent. In brief, RNA was reversed to cDNA for constructing the library. Then, RNA sequencing was conducted on the cDNA library. The raw reads were filtered, and clean reads were mapped to GRCh37 whole genome using STAR2.7.3a. The raw gene counts was calculated using htseq-count. The gene expression level (FPKM) was calculated according to the RSEM, and the data were analyzed.

### Western Blot

Cells were harvested and lysed in SDS sample buffer. The same amount of protein samples was electrophoresed through 8–10% SDS-PAGE and transferred to a nitrocellulose blotting membrane. Following 5% non-fat milk or BSA blocking, these membranes were incubated at 4°C overnight with primary antibodies. Detailed information regarding antibodies were as follows: ACTIN, TUBULIN as internal control (1:2,000; Hua An Biotechnology, Hangzhou, China); Phospho-Rpb1 CTD (Ser2) (#13499, 1:1,000; CST); Phospho-Rpb1 CTD (Ser5) (#13523, 1:1,000; CST), Phospho-Rpb1 CTD (Ser7) (#13780, 1:1,000; CST); Rpb1 CTD (1:1,000; AM39097); BCL2 (1:1,000; Biotime); caspase 3 (ab13847, 1:1,000; Abcam); cleaved caspase 3 (#9661, 1:1,000; CST); HK1 (#2024T, 1:1,000; CST); LDHA (#3582, 1:1,000; CST); c-MYC (#5605, 1:1,000; CST, Danvers, MA, USA); Flag (0912-1, 1:1,000; Hua An Biotechnology, Hangzhou, China); PFKP (#12746, 1:1,000, CST); PKM2 (#4053, 1:1,000, CST). After incubation with the fluorescence-labeled secondary antibody, fluorescence signals were analyzed using the BIO-RAD ChemiDocTM MP Imaging System.

### Apoptosis Assay

Cells were incubated with THZ1 or DMSO at indicated concentrations and harvested after 6–24 h. After washing with phosphate-buffered saline (PBS) two times, cell apoptosis was measured using the Annexin-V apoptosis detection kit (BD Bioscience, San Jose, CA, USA) according to the manufacturer's protocol. The percentage of Annexin-V-positive cells was detected by flow cytometry, and the data were analyzed using FlowJo Version 10.0 software.

### Cell Cycle Analysis

After treatment for 2–24 h with THZ1 or DMSO, EdU was added into the cells, and the cells were incubated for 2 h. Cell proliferation was conducted using Click-iT EdU flow cytometry assay kit (Beyotime) according to the manufacturer's protocol. The stained cells were then analyzed using a flow cytometer.

### Measurements of Metabolites

After cells were treated with 500 nM THZ1 or DMSO in RPMI 1640 complete medium for 24 h, 2-NBDG (Invitrogen) was used to detect the glucose uptake ability of the cells. Other metabolic dyes, such as mitochondrial membrane potential probe MitoTrackerTM orange (Life Technologies, M7511), total ROS probe DCFDA (Life Technologies, C369), and MitoSOXTM Red mitochondrial superoxide indicator (Life Technologies, 2189134) were used to stain the samples. Then, the samples were analyzed by flow cytometry. An enhanced ATP assay kit (Beyotime Biotechnology) was used to determine the intracellular ATP of cells.

### Metabolite Analysis

A total of 2 × 10^6^ cells were harvested after treatment with drugs for 24 h and washed with pre-cooled PBS. Cells were cracked in ice-cold 80% methanol and centrifuged at 1,500 rpm for 10 min to obtain the supernatant. Finally, the supernatant was analyzed by liquid chromatography–tandem mass spectrometry (LC–MS/MS).

### Statistical Analysis

Statistical analysis was conducted using GraphPad Prism 7.0 (GraphPad Software). Data were presented as mean ± SD of three independent experiments. Differences between samples were analyzed using two-tailed Student's *t*-test. The results with values of *P* < 0.05 were considered statistically significant.

## Results

### THZ1 Arrests the Cell Cycle of B-ALL Cell Lines *in vitro*

**Figure 1:**
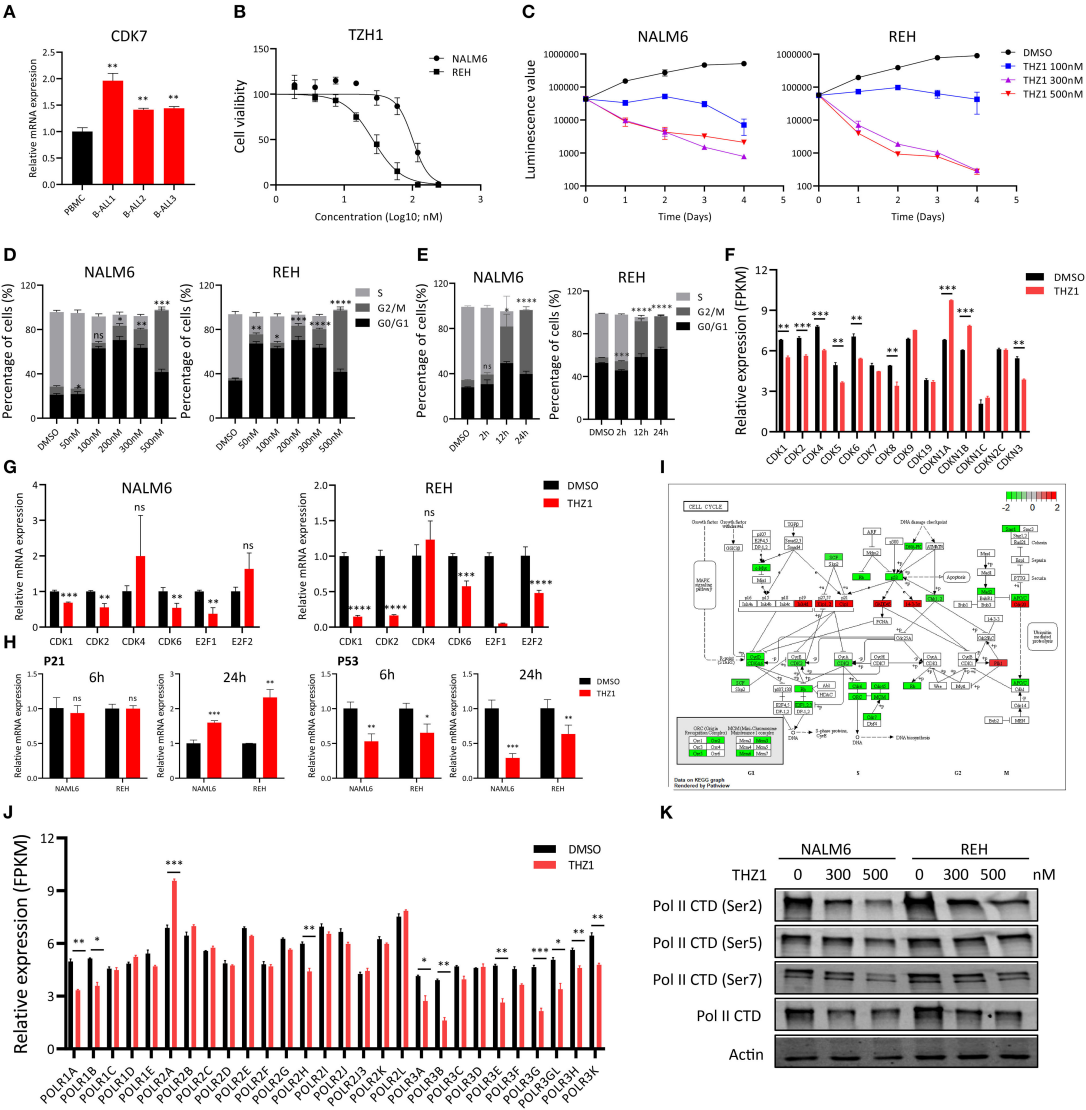
THZ1 inhibits cell proliferation in B-ALL cells. **(A)** Relative mRNA expression of CDK7 in primary B-ALL sample and human PBMCs. **(B)** Drug sensitivity assay of NALM6 and REH cells after treatment with different concentrations of THZ1 for 72 h. **(C)** Cell count assay of NALM6 and REH cells after treatment with different concentrations of THZ1. **(D)** EdU-labeled cell cycle of B-ALL cell lines was analyzed by flow cytometry after treatment with different concentrations of THZ1 for 24 h. **(E)** Cell cycle of B-ALL cell lines was analyzed by flow cytometry after treatment with THZ1 for 2, 12, and 24 h. **(F)** CDKs expression was detected by RNA-SEQ in THZ1- and DMSO-treated NALM6 cells. **(G)** The mRNA expression of CDKs and E2F family was measured by qRT-PCR in THZ1- and DMSO-treated B-ALL cells. **(H)** The mRNA expression of p21 and p53 was measured by qRT-PCR in THZ1- and DMSO-treated B-ALL cells. **(I)** Pathview analysis of down and upregulated cell cycle-related genes in THZ1-treated NALM6 cells. **(J)** The mRNA expression of the POLR family was assessed by RNA-SEQ in THZ1- and DMSO-treated NALM6 cells. **(K)** The protein expression of Ser2, Ser5, Ser7, and total RNAPII was determined by Western blot in THZ1- and DMSO-treated B-ALL cells. Values were shown as mean ± SEM. ^*^p < 0.05, ^**^p < 0.01, ^***^p < 0.001, and ^****^p < 0.0001.

To investigate the anti-proliferative effect of CDK7 inhibitor on the B-ALL cells, we first determined the CDK7 expression in B-ALL patients' sample and human peripheral blood mononuclear cells (PBMCs). The elevated CDK7 expression was observed in primary B-ALL cells (Figure 1A). Then, the cell viability of B-ALL cell lines, NALM6 and REH, was measured by ATP detection kit after treatment with a gradient concentration of THZ1 for 72 h. The half-maximal inhibitory concentration (IC50) values of NALM6 and REH were 101.2 and 26.26 nM, respectively (Figure 1B). Cell count assay showed that 100 nM of THZ1 evidently inhibited the growth of B-ALL cells (Figure 1C). Subsequently, cell proliferation was also assessed by staining with EdU, and the results showed that THZ1 treatment dramatically arrested B-ALL cells at the G2/M phase of cell cycle in a concentration and time-dependent manner (Figures 1D,E). In addition, we performed RNA sequencing (RNA-SEQ) and qRT-PCR to detect the expression of cell cycle-related genes. The RNA-SEQ results displayed that THZ1 treatment significantly downregulated the expression of cell proliferation-related genes, such as CDK1, CDK2, CDK6, and CDK8, and upregulated the expression of cell cycle arrest-related genes, such as CDKN1A and CDKN1B (Figure 1F). These results were also confirmed by qRT-PCR (Figures 1G,H). Pathway analysis demonstrated that the downregulated cell proliferation-related genes were associated with the cell cycle of B-ALL cells after treatment with THZ1 (Figure 1I). The anti-proliferative effects of THZ1 were verified through phosphorylating serine 2, 5, and 7 in the CTD of RNA Pol II in B-ALL cells but not the mRNA expression of CDK7 (Figures 1F,J,K). Thus, these data indicated that THZ1 covalently bound to CDK7 and inhibited the downstream genes that were regulated by CDK7 transcription complex, which was consistent with previous reports (11, 13, 20, 27).

### THZ1 Induces the Cell Apoptosis of B-ALL Cell Lines *in vitro*

**Figure 2:**
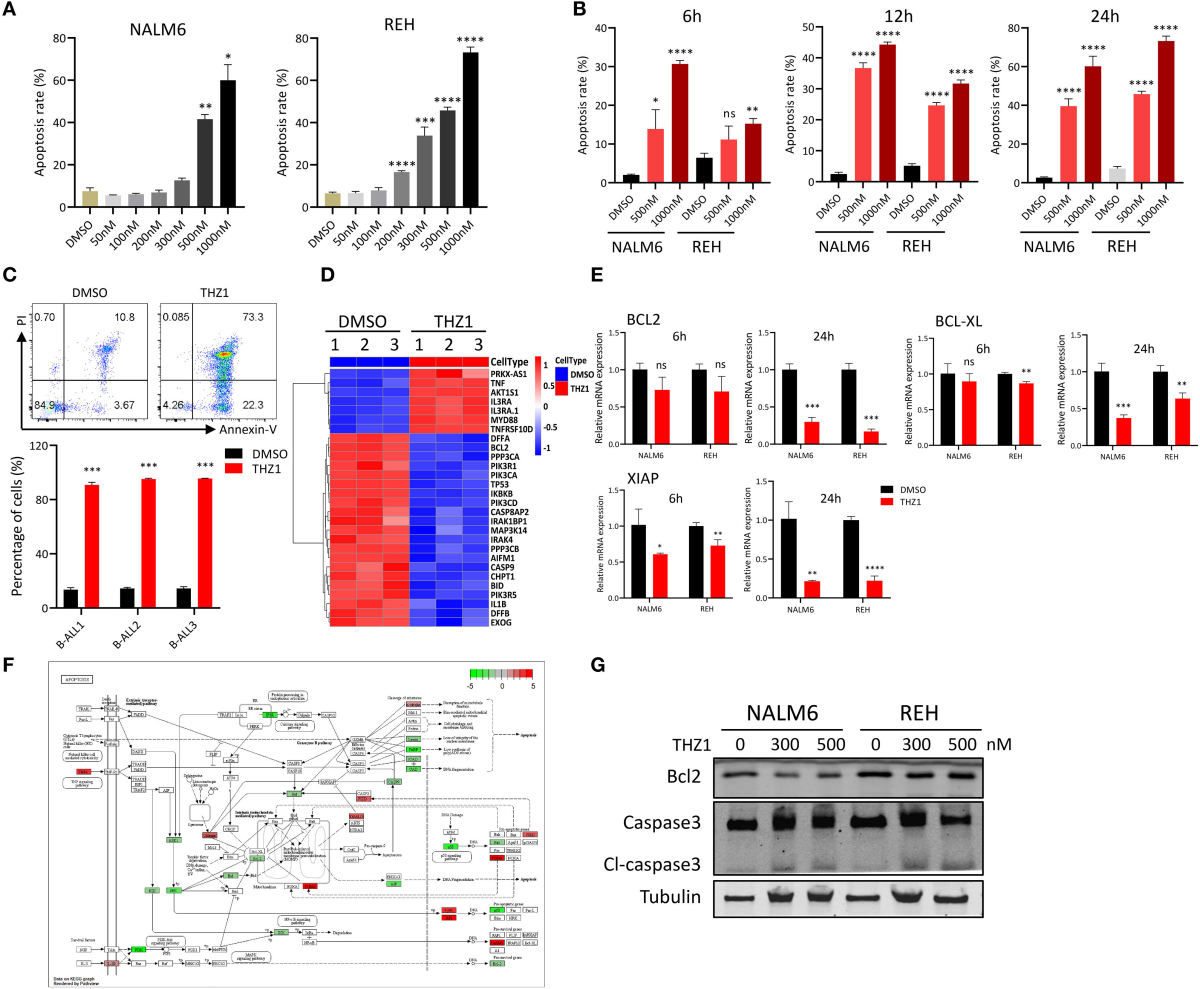
THZ1 induces apoptosis in B-ALL cells. **(A)** Cell apoptosis of NALM6 and REH cells was analyzed by flow cytometry using Annexin V and PI double staining after treatment with different concentrations of THZ1. **(B)** Cell apoptosis of NALM6 and REH cells was analyzed by flow cytometry after treatment with THZ1 for 6, 12, and 24 h. **(C)** Cell apoptosis of primary B-ALL cells was analyzed by flow cytometry using Annexin V and PI double staining after treatment with THZ1 for 24 h. **(D)** Heatmap of cell apoptosis-related genes in THZ1- and DMSO-treated NALM6 cells. **(E)** The mRNA expression of BCL2, BCL-XL, and XIAP was measured by qRT-PCR in THZ1- and DMSO-treated B-ALL cells. **(F)** Pathview analysis of down and upregulated cell apoptosis-related genes in THZ1-treated B-ALL cells. **(G)** Anti-apoptotic BCL2 and apoptotic Caspase 3 proteins of B-ALL cells were detected by Western blot. B-ALL cells were treated with THZ1 for 24 h. Values were shown as mean ± SEM. ^*^p < 0.05, ^**^p < 0.01, ^***^p < 0.001, and ^****^p < 0.0001.

Previous studies indicated that different THZ1 concentrations have distinct cytotoxic effects on tumor cells, with low THZ1 concentrations inhibiting cell proliferation and high THZ1 concentrations inducing cell apoptosis (13, 14). Cell apoptosis was tested by Annexin-V and PI double staining to evaluate whether THZ1 could induce cell death of B-ALL cells in a high concentration. Exposure of B-ALL cells to increasing concentrations of THZ1 for 24 h variably reduced cell survival, of which only high THZ1 concentrations induced cell apoptosis (Figure 2A). In a high concentration, THZ1 treatment promoted apoptotic death within 6 h (Figure 2B), indicating that THZ1 also induced the cell apoptotic death of B-ALL. The cell apoptosis of B-ALL samples from patients was also determined, and the result exhibited that the apoptotic rates increased in THZ1-treated sample compared with that of DMSO-treated sample (Figure 2C, Supplementary Table 1). We further checked the expression of apoptosis- and anti-apoptosis-related genes. THZ1 treatment significantly downregulated the expression of anti-apoptotic genes, such as BCL2 and BCL-XL (Figures 2D–F), which are the key regulators in the mitochondrial apoptotic pathway. In addition, the protein expression levels of anti-apoptotic gene BCL2 and apoptotic gene Caspase 3 were determined by Western blot. The results demonstrated that THZ1 treatment remarkably decreased BCL2 and increased cleaved caspase 3 of B-ALL cells (Figure 2G). Collectively, these data suggested that CDK7 inhibitor THZ1 induced cell apoptosis of B-ALL *in vitro* by activating the mitochondrial apoptotic signal pathway.

### THZ1 Disrupts the Cellular Metabolic Pathways of B-ALL Cells *in vitro*

**Figure 3:**
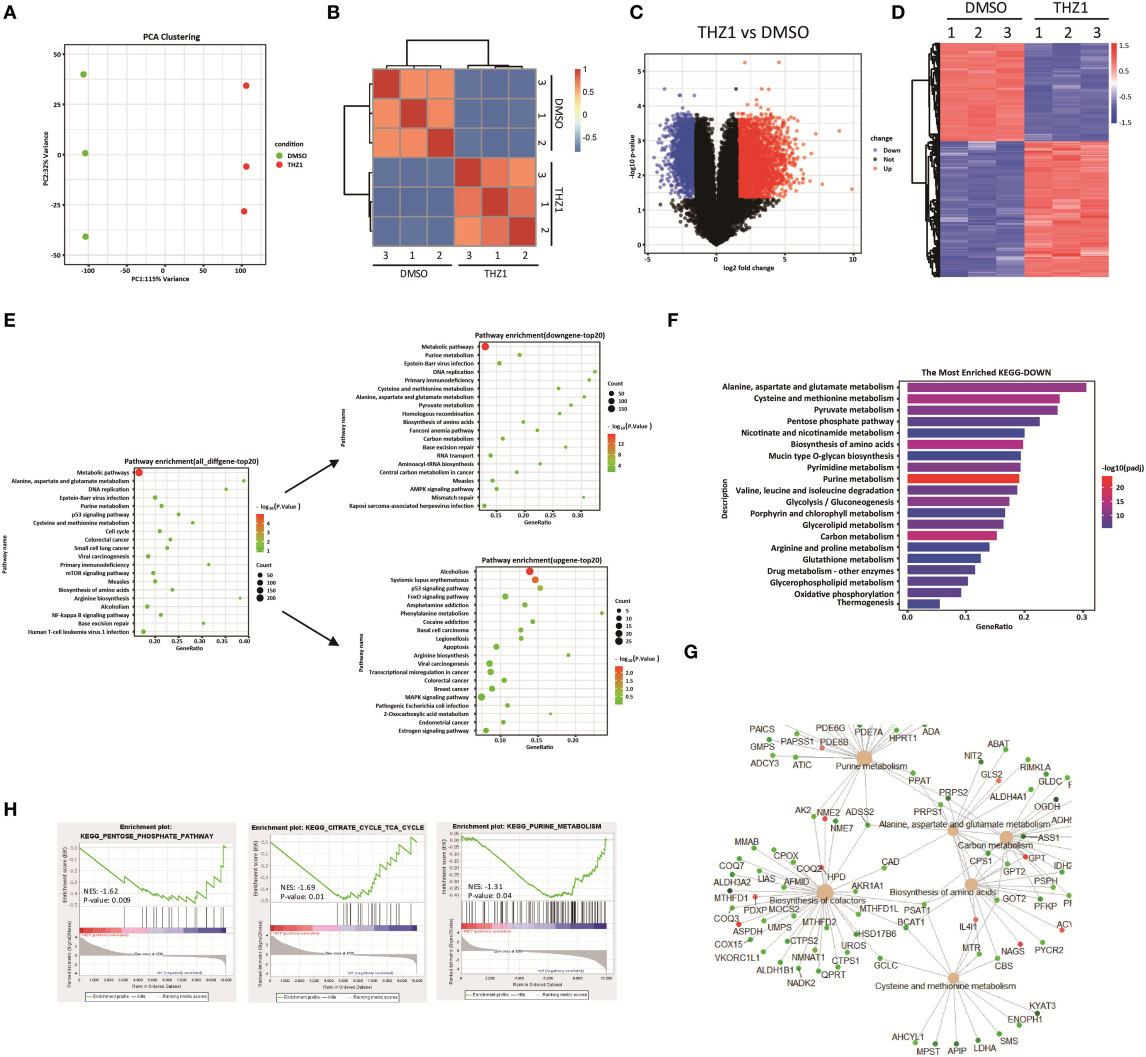
THZ1 perturbs the cellular metabolic pathways of B-ALL cells. **(A,B)** Principal component analysis and correlation analysis of the RNA-SEQ data in THZ1- and DMSO-treated NALM6 cells. **(C,D)** Volcano plot and heatmap of differentially expressed genes by a log2-fold change ≥ 1.5 or ≤ −1.5 (*p* < 0.05) in THZ1- and DMSO-treated NALM6 cells. **(E)** Pathway analysis of upregulated and downregulated genes (*p* < 0.05) in THZ1- and DMSO-treated NALM6 cells. **(F)** KEGG pathway analysis of downregulated metabolic genes (*p* < 0.05) in THZ1- and DMSO-treated NALM6 cells. **(G)** KEGG pathway analysis of downregulated metabolic genes (*p* < 0.05) in THZ1- and DMSO-treated NALM6 cells. **(H)** Gene set enrichment analysis of the genes related to glucose, amino acid, and nucleotide metabolism. B-ALL cells were treated with THZ1 for 24 h.

During tumor progression, tumor cells reprogramed their metabolic state from catabolism to anabolism for uncontrolled cell proliferation (15, 16). Previous study reported that CDK7 inhibition suppresses human non-small-cell lung cancer cells through interference with cancer metabolism (25). However, whether CDK7 inhibitors interfere with the cellular metabolism of B-ALL cells remains unknown. In addressing this question, the RNA-SEQ data of THZ1- and DMSO-treated cells were re-analyzed by correlation analysis and principal component analysis, and the results exhibited that THZ1- and DMSO-treated cell populations were fairly clustered (Figures 3A,B). THZ1 treatment globally changed the transcript profile of B-ALL cells, with 2,614 genes upregulated and 1,892 genes downregulated when compared with those of DMSO-treated cells (Figures 3C,D). We then performed Kyoto Encyclopedia of Genes and Genomes (KEGG) analysis to enrich the pathways of upregulated and downregulated genes. THZ1 treatment significantly disturbed the mRNA expression of the metabolic pathways, DNA replication, p53 signaling pathway, and cell cycle (Figure 3E), where the top 20 downregulated genes were clustered such as the metabolic pathways, purine metabolism, DNA replication, and carbon metabolism (Figure 3E). Furthermore, the downregulated metabolic pathways were subdivided into pentose phosphate pathway (PPP), pyrimidine and purine metabolism, and TCA cycle (Figures 3F–H). Our data indicated that THZ1 perturbed the cellular metabolic pathways of B-ALL cells *in vitro*.

### THZ1 Suppresses the Cellular Metabolism of B-ALL Cells

**Figure 4:**
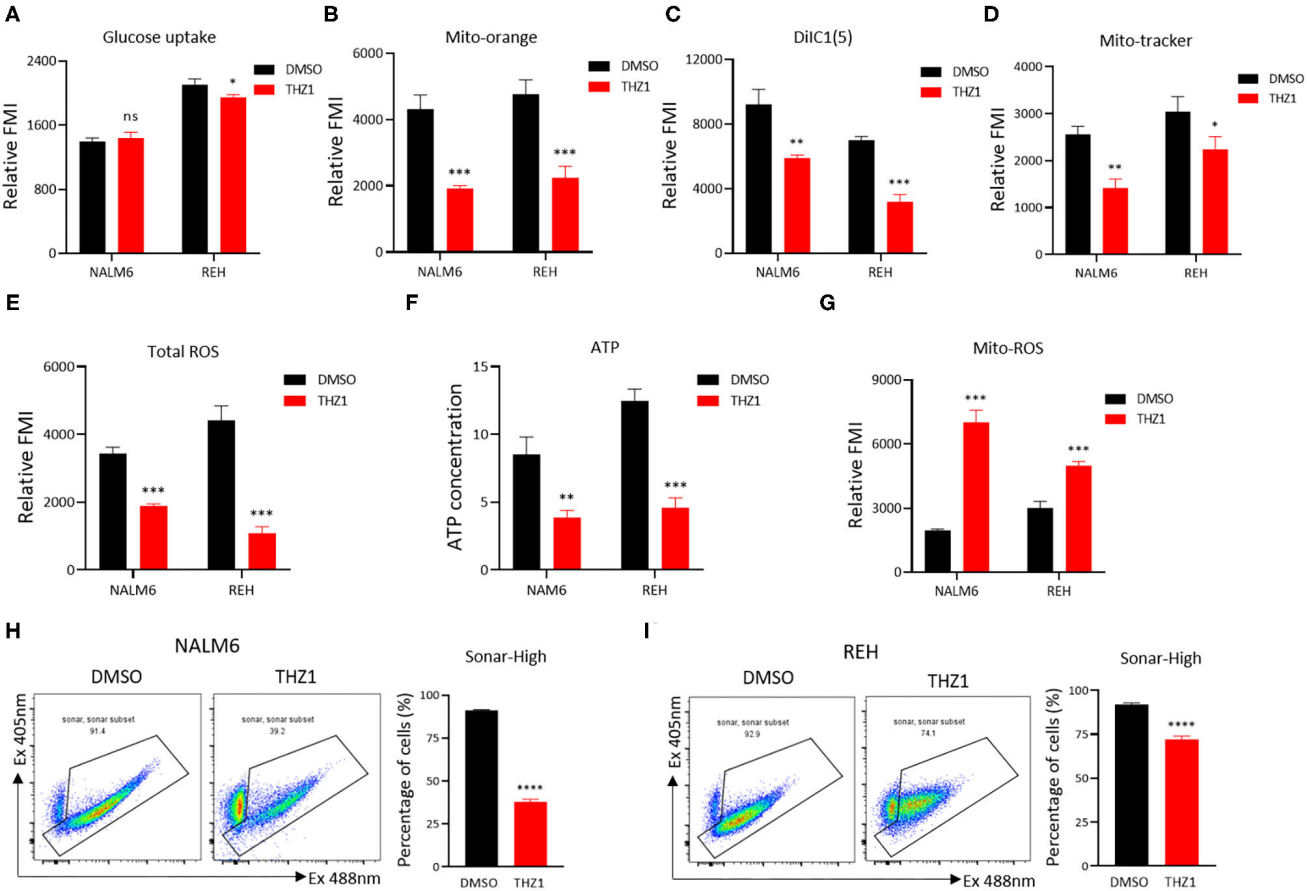
THZ1 suppresses the cellular metabolism of B-ALL cells. **(A)** The glucose uptake of NALM6 and REH cells was detected by flow cytometry. **(B–E)** The mitochondrial membrane potential (MMP), mitochondrial mass (MM), and total ROS of B-ALL cells were detected by flow cytometry. **(F)** The quantification of intracellular ATP in B-ALL cells was assessed using the ATP determination kit. **(G)** The mitochondrial ROS (Mito-ROS) of B-ALL cells were detected by flow cytometry. **(H,I)** SoNar-low or -high B-ALL cells were measured by flow cytometry with 405 and 488 nm excitation. B-ALL cells were treated with THZ1 for 24 h. Values were shown as mean ± SEM. ^*^p < 0.05, ^**^p < 0.01, ^***^p < 0.001, and ^****^p < 0.0001.

A recent study identified that CDK7 is a key regulator of glucose consumption, and CDK7 inhibitors block glucose consumption in lung cancer cells (26). The glucose uptake of B-ALL cells was detected by incubating fluorescence-labeled 2-deoxy-glucose analog (2-DG) to prove that THZ1 treatment had an inhibitory effect on the cellular metabolism of B-ALL cells. However, THZ1 treatment did not significantly decreased the glucose uptake activities in B-ALL cells (Figure 4A). Nevertheless, the results of metabolic analysis showed that THZ1 treatment markedly reduced the mitochondrial membrane potential (MMP), mitochondrial mass (MM), total reactive oxygen species, and ATP content in B-ALL cells (Figures 4B–F). Notably, the mitochondrial reactive oxygen species (Mito-ROS) was increased in THZ1-treated cells (Figure 4G), implying that Mito-ROS activated the mitochondrial apoptotic pathway. SoNar probe, a metabolic sensor, was used to dynamically monitor the metabolic change, and the SoNar-high cells preferred glycolysis (28, 29). The results of flow cytometry revealed that THZ1 treatment dramatically reduced the ratios of SoNar-high cells (Figures 4H,I). Therefore, THZ1 suppressed the cellular metabolism of B-ALL cells by restraining the downstream of glucose uptake.

### THZ1 Restrains the Production of Cellular Glycolytic and Nucleotide Intermediates in B-ALL Cells

**Figure 5:**
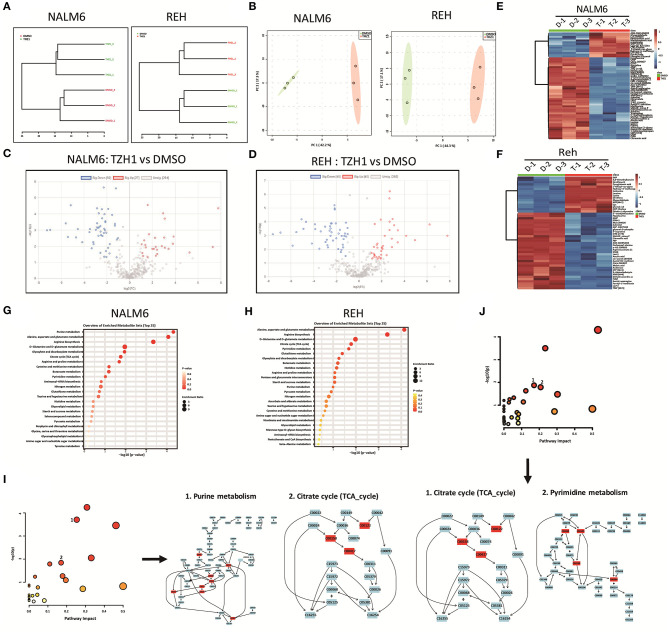
THZ1 blocks the production of metabolic intermediates in B-ALL cells. **(A,B)** Correlation analysis and principal component analysis of the metabonomic data in THZ1- and DMSO-treated NALM6 and REH cells. **(C,D)** Volcano plot of differentially metabolic intermediates by a log2-fold change ≥ 1.0 or ≤ −1.0 (*p* < 0.05) in THZ1- and DMSO-treated NALM6 and REH cells. **(E,F)** Heatmap of differentially metabolic intermediates by a log2-fold change ≥ 1.0 or ≤ −1.0 (*p* < 0.05) in THZ1- and DMSO-treated NALM6 and REH cells. **(G,H)** Pathway analysis of upregulated and downregulated metabolic intermediates (*p* < 0.05) in THZ1- and DMSO-treated NALM6 and REH cells. **(I,J)** Pathway analysis of downregulated metabolic intermediates (*p* < 0.05) in THZ1- and DMSO-treated NALM6 and REH cells. B-ALL cells were treated with THZ1 for 24 h.

The intermediates of total energy metabolism in THZ1-treated B-ALL cells were measured by LC–MS/MS to further confirm that CDK7 inhibitor THZ1 blocked the cellular metabolism of B-ALL cells *in vitro*. Correlation analysis and principal component analysis revealed that the three THZ1- and DMSO-treated cell populations were legitimately clustered (Figures 5A,B). THZ1 treatment considerably altered the metabolic intermediate profile of B-ALL cells, leading to an increase of 27 metabolites and a decreased of 50 metabolites in NALM6 and an increase of 40 metabolites and a decrease of 46 metabolites in REH (Figures 5C–F). The rising and falling of metabolic intermediates enriched purine and pyrimidine metabolism; glycolytic and TCA cycle metabolism (Figures 5G,H), which is consistent with the results of RNA-SEQ. In addition, THZ1 treatment significantly led to the drop of the metabolic intermediate levels of purine and pyrimidine metabolism, such as guanosine monophosphate and cytidine triphosphate; and TCA cycle, such as fumaric acid and citric acid (Figures 5I,J, Supplementary Table 2). Thus, THZ1 restrained the cellular metabolic intermediates of B-ALL cells *in vitro*.

### THZ1 Inhibits Cell Metabolism of B-ALL by Downregulating the Expression of c-MYC-Mediated Metabolic Enzymes

**Figure 6:**
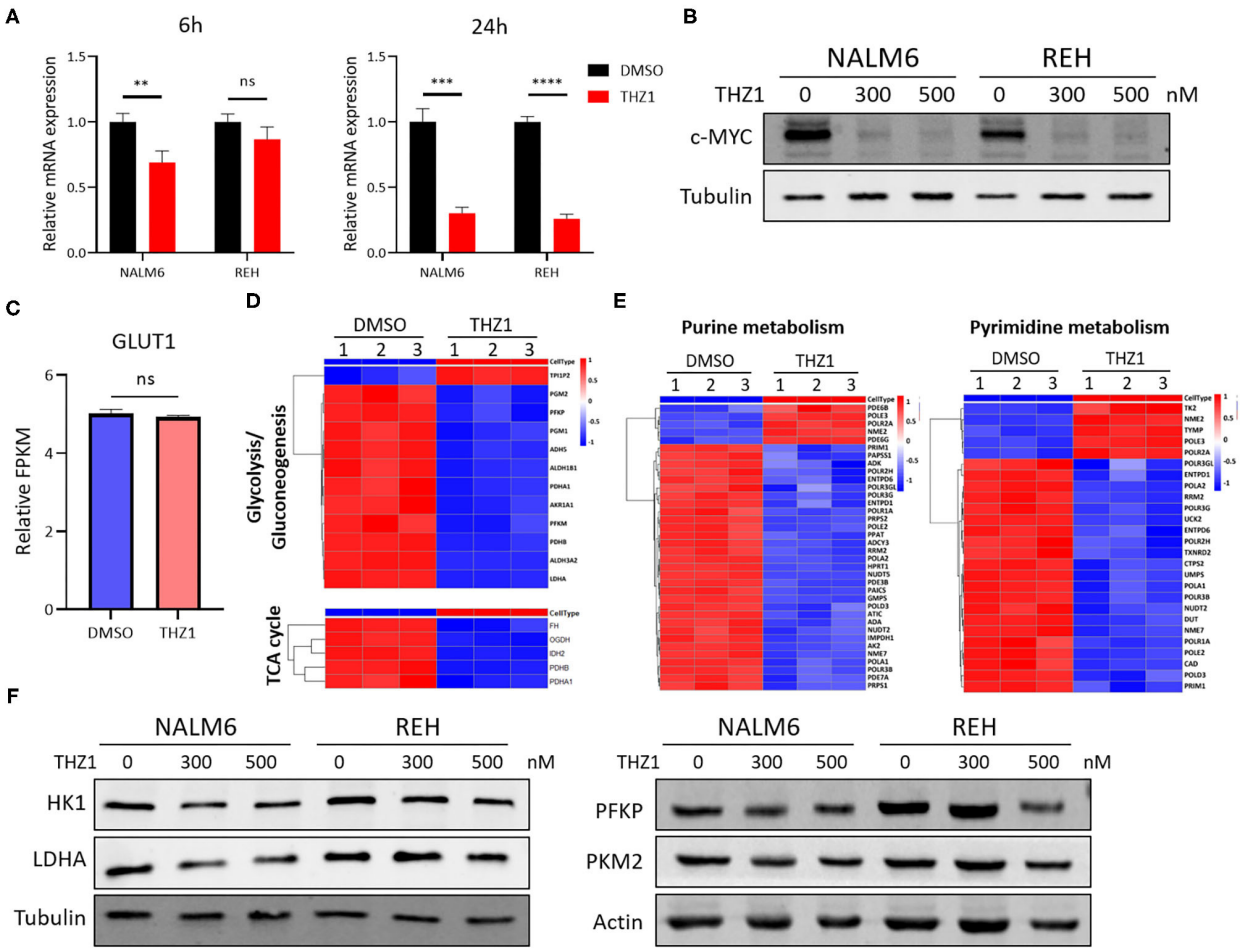
THZ1 disturbs the cellular metabolism of B-ALL cells by downregulating c-MYC-mediated metabolic enzymes. **(A)** Relative mRNA expression of c-MYC was measured by qRT-PCR in THZ1- and DMSO-treated NALM6 and REH cells. **(B)** Protein expression of c-MYC was determined by Western blot in THZ1- and DMSO-treated NALM6 and REH cells. **(C)** Relative mRNA expression of GLUT1 was measured by RNA-SEQ in THZ1- and DMSO-treated NALM6 cells. **(D,E)** Heatmap of glucose, amino acid, and nucleotide metabolism-related genes was analyzed by RNA-SEQ in THZ1- and DMSO-treated NALM6 cells. **(F)** Protein expression levels of glucose and nucleotide metabolism-related genes, such as HK1, LDHA, and PFKP, were detected by Western blot in THZ1- and DMSO-treated NALM6 and REH cells. B-ALL cells were treated with THZ1 for 24 h. Values were shown as mean ± SEM. ^**^p < 0.01, ^***^p < 0.001, and ^****^p < 0.0001.

Selectively targeting CDK7 has been proven as a useful therapy for cancers that are driven by MYC amplification (20, 22). THZ1 treatment significantly inhibits the growth of tumors with concurrent abrogation of MYC expression (21, 22). Studies have shown that MYC facilitates the anabolism of cancer cells by modulating the expression of multiple metabolic enzyme genes, such as GLUT1, PKM2, and LDHA (30, 31). In verifying whether THZ1 induces cell apoptosis by repressing the expression of c-MYC-mediated metabolic genes, the mRNA and protein expression of c-MYC was measured in THZ1-treated B-ALL cells. THZ1 treatment significantly suppressed the expression of c-MYC (Figures 6A,B). We first detected the expression of GLUT1, a main glucose transporter, and found that THZ1 treatment did not significantly downregulated the expression of GLUT1 (Figure 6C), which was consistent with the result that THZ1 did not affect the glucose uptake activities (Figure 4A). Nevertheless, the results of RNA-SEQ data showed that THZ1 treatment markedly downregulated the expression of rate-limiting enzymes of glycolytic metabolism, such as PFKP, PDHA, and LDHA, and TCA cycle metabolism, such as FH and IDH2 (Figure 6D). Moreover, THZ1 treatment significantly decreased the expression of rate-limiting enzymes of purine and pyrimidine metabolism (Figure 6E). In addition, the protein expression levels of the rate-limiting enzymes in the glycolytic pathway were detected by Western blot. We found that THZ1 treatment markedly downregulated the protein expression levels of rate-limiting enzymes, such as HK1, LDHA, and PFKP (Figure 6F). Collectively, these data suggested that THZ1 disturbed the cellular metabolism of B-ALL cells by inhibiting the expression of metabolic enzymes.

### THZ1 Enhances the Cell Apoptosis of c-MYC-Overexpressing B-ALL Cells

**Figure 7:**
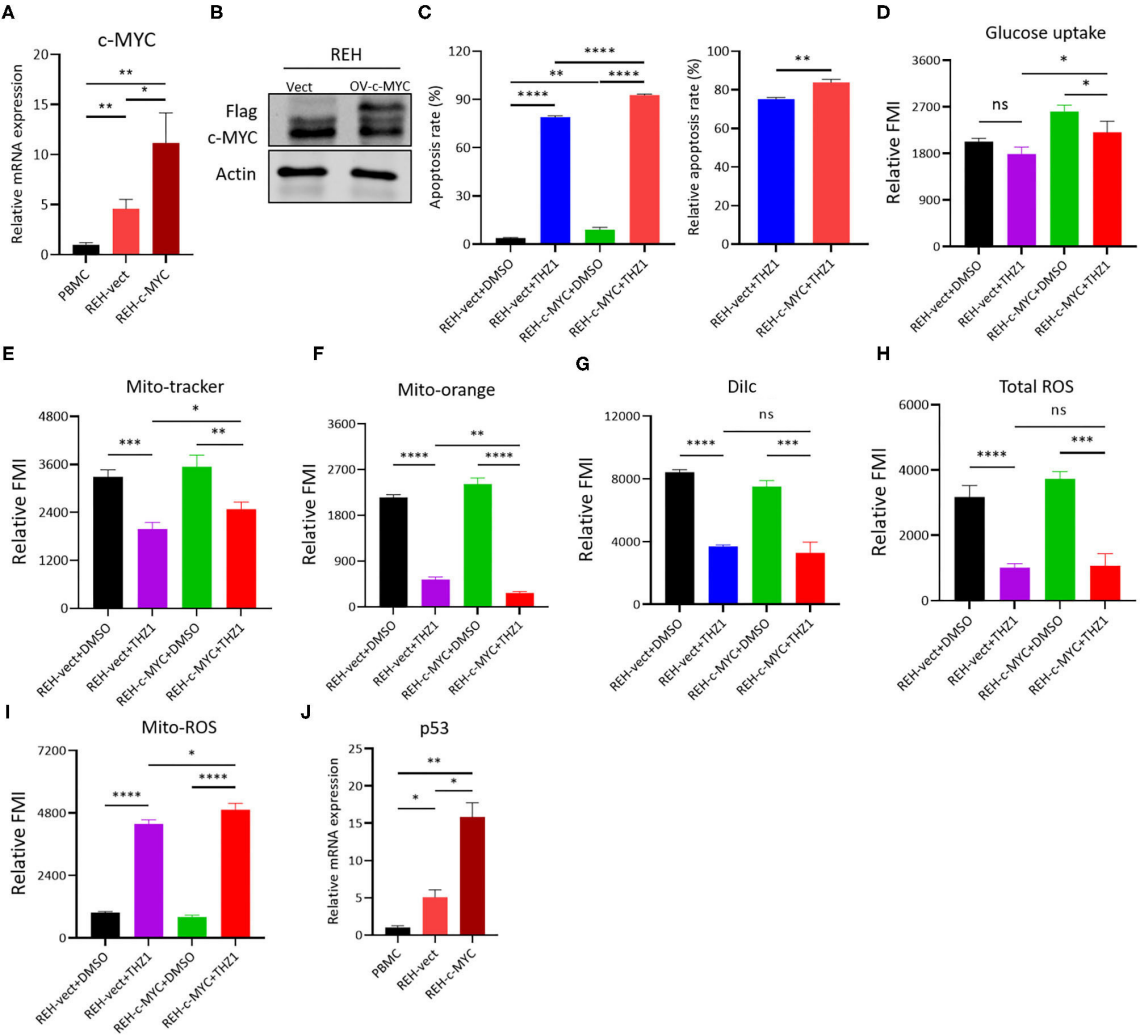
THZ1 enhances cell apoptosis in c-MYC-overexpressed B-ALL cells. **(A,B)** Overexpressed mRNA and protein levels of c-MYC in REH cells was detected by qRT-PCR and Western blot, respectively. **(C)** Annexin V- and PI-labeled cell apoptosis of c-MYC-overexpressed REH cells were analyzed by flow cytometry. **(D)** Glucose uptake of vector and c-MYC-overexpressed REH cells was analyzed by flow cytometry. **(E–H)** Mitochondrial membrane potential (MMP), mitochondrial mass (MM), and total ROS of vector and c-MYC-overexpressed REH cells were analyzed by flow cytometry. **(I)** The mitochondrial ROS (Mito-ROS) of vector and c-MYC-overexpressed REH cells were analyzed by flow cytometry. **(J)** The mRNA expression of p53 in vector and c-MYC-overexpressed REH cells were determined by qRT-PCR. B-ALL cells were treated with THZ1 for 24 h. Values were shown as mean ± SEM. ^*^p < 0.05, ^**^p < 0.01, ^***^p < 0.001, and ^****^p < 0.0001.

The c-MYC was over-expressed on REH cells by lentivirus infection and Western blot to verify whether the THZ1-induced reduction of cellular metabolism in B-ALL cells was regulated by c-MYC, and the result confirmed that the mRNA and protein levels of c-MYC were upregulated in REH cells (Figures 7A,B). Then, the c-MYC over-expressing REH cells were treated with THZ1. Unexpectedly, the ratios of cell apoptosis were evidently increased in c-MYC over-expressing REH cells after intervention with THZ1 (Figure 7C). Meanwhile, the levels of glucose uptake and MM were partially increased in c-MYC-overexpressing REH cells after treatment with THZ1 (Figures 7D,E). The MMP was evidently reduced in c-MYC-overexpressing REH cells after treatment with THZ1 (Figures 7F,G). THZ1 treatment did not affect the level of total ROS (Figure 7H), but this treatment boosted more Mito-ROS production in overexpressed c-MYC REH cells (Figure 7I), indicating that THZ1 resulted in the activation of the Mito-ROS mitochondrial apoptotic pathway. Recently, two studies have shown that CDK7 inhibitors augmented anti-tumor activity through activating the p53 transcriptional program in tumor cells (32, 33). We examined the mRNA expression of p53 gene by qRT-PCR to test whether over-expressing c-MYC in REH cells resulted in the upregulation of p53. Our data demonstrated that the mRNA expression of p53 was robustly increased in overexpressed c-MYC REH cells (Figure 7J). These findings implied that CDK7 inhibitor enhanced the cell apoptosis of c-MYC-overexpressing B-ALL cells by partly increasing the p53 expression because of c-MYC over-expression.

## Discussion

In the present study, we showed that THZ1 arrested the cell cycle of B-ALL cells *in vitro* in a low concentration, while inducing the apoptosis of B-ALL cells *in vitro* in a high concentration by activating the apoptotic pathways. In addition, RNA-SEQ results revealed that THZ1 disrupted the cellular metabolic pathways of B-ALL cells. Moreover, THZ1 suppressed the cellular metabolism and blocked the production of cellular metabolic intermediates in B-ALL cells. Mechanistically, THZ1 inhibited the cellular metabolism of B-ALL by downregulating the expression of c-MYC-mediated metabolic enzymes, such as HK1, PFKP, PKM2, and LDHA.

Transcription addiction contributes to the uncontrolled cell proliferation of tumors. Therefore, therapeutically targeting of transcription factors is an attractive target for tumor therapy (34). CDK7 is a transcription addiction factor that is upregulated in various types of tumor and correlated with prognosis of patients (5). In the present study, we first determined that CDK7 was upregulated in B-ALL cell lines, indicating that CDK7 might be a transcription factor for B-ALL cell proliferation. THZ1, a covalent CDK7 inhibitor, treats tumor types that are dependent on transcription addiction (7, 35). Therefore, we evaluated the therapeutic potential of THZ1 as a candidate drug for the treatment of B-ALL. Our results showed that THZ1 arrested B-ALL cells in the G2/M phase of cell cycle in a low concentration, with the downregulation of cyclins and CDKs. Moreover, THZ1 induced the apoptosis of B-ALL cells in a high concentration. We also performed apoptosis assay to test the effect of THZ1 on normal human peripheral blood mononuclear cells (PBMCs). Admittedly, we found that high concentration of THZ1, to a large extent, induced apoptosis of PBMCs (data not shown), indicating that THZ1 may lead to severe side effects, such as myelosuppression. The possible reason is that the cellular uptake and absorption rate of THZ1 might be low in leukemic cells. We further discovered that THZ1 treatment remarkably decreased anti-apoptotic protein BCL2 and increased cleaved caspase 3 in B-ALL cells, suggesting that the therapeutic effect of CDK7 inhibitor was dependent on triggering programmed cell death. Our findings are consistent with the results that are reported in solid tumors (8, 9, 11, 13, 14). We also confirmed that the inhibitory effect of THZ1 on B-ALL cells was associated with the reduction of CTD phosphorylation of RNA Pol II at Ser-2, Ser-5, and Ser-7. These data were consistent with the results of previous studies, which showed that CDK7 was closely involved in cell cycle and transcription (36).

Tumor cells reprogrammed their metabolism from catabolism to anabolism to support rapid cell proliferation. The cellular metabolism of cancer cells is characterized by enhanced glycolysis, accompanied with increased rates of lactate and ATP production, nucleotide synthesis, and biosynthesis of lipids and other macromolecules (15, 17). Recently, studies have found that B-ALL cells heavily rely on the ATP supply and pyruvate and TCA cycle metabolites derived from glycolysis by repressing rate-limiting PPP enzymes (37–39). CDKs promote the cell cycle progression of tumor cells by regulating the catalytic activity of metabolic rate-limiting enzymes (18, 40). However, the effect of CDK7 inhibitors on the cellular metabolism of leukemia is unclear. In accordance with previous studies indicating that CDK7 inhibitors induce cell cycle arrest and apoptosis of tumor cells via blocking the glucose consumption or interfering with cancer metabolism (25, 26). Our RNA-SEQ data also showed that THZ1 disrupted the cellular metabolic pathways of B-ALL cells, particularly the glycolysis and nucleotide synthesis pathway. Thus, we proposed that CDK7 inhibitors resulted in the apoptosis of B-ALL cells by perturbing the cellular metabolism. We also discovered that THZ1 could significantly restrain the glycolysis of B-ALL cells by inhibiting glucose absorption and metabolic process, thereby reducing the metabolic intermediates, such as lactate and ATP, which were the main materials and energy sources for cell synthesis (41, 42). Based on the results of RNA-SEQ, THZ1 downregulated the expression of many key enzymes of the cellular metabolism, such as HK1, PFKP, and LDHA. These results provide additional evidence that cell cycle progression is inextricably bound up with cellular metabolism, supporting the conclusion that CDK6 and CDK8 play a role in regulating glucose consumption and glycolysis (18, 19).

More recently, several studies have reported that THZ1 suppresses cell growth and induces cell apoptosis of tumors by downregulating the c-MYC expression (13, 20, 43). A new study has revealed that THZ1 enhances anti-PD-1 therapy efficacy via the c-MYC-mediated signaling pathway in non-small cell lung cancer (44). Consistent with these findings, we uncovered that THZ1 considerably diminished the mRNA levels and protein expression of c-MYC in B-ALL cells. c-MYC contributes to the glucose metabolic reprogramming of tumor cells by regulating the expression of different target genes (45, 46). Thus, we infer that the cellular metabolism of B-ALL cells is disturbed by CDK7 inhibitor THZ1 through the reduction of the c-MYC level. Subsequently, we performed a rescue experiment by overexpressing c-MYC in REH cells, and the cells were treated with THZ1. Consequently, THZ1 treatment enhanced cell apoptosis in over-expressed c-MYC REH cells. Meanwhile, we observed that THZ1 treatment increased the glucose uptake and quantity of the mitochondria, while decreasing MMP in REH cells with overexpressed c-MYC. However, THZ1 treatment boosted more mito-ROS production in overexpressed c-MYC REH cells, indicating that THZ1 resulted in the activation of the mito-ROS mitochondrial apoptotic pathway. CDK7 inhibitors augmented the anti-tumor activity through activating the p53 transcriptional program in tumor cells (32, 33). In addition, we examined the expression of p53 gene to explore the mechanism of overexpressing c-MYC REH cells sensitive to THZ1 treatment. Our data demonstrated that the mRNA expression of p53 was robustly increased in overexpressed c-MYC REH cells. Our study further supports that MYC-amplified tumors are highly vulnerable to the treatment of CDK7 inhibitors (20–22). Whether MYC-amplified B-ALL cells are sensitive to the treatment of CDK7 inhibitors requires further exploration. In addition, whether c-MYC regulated the expression of the key enzymes through directly binding to the promoters of glycolytic genes requires further exploration.

## Conclusion

Our data demonstrated that THZ1 induced the apoptosis of B-ALL cells *in vitro* in a high concentration. THZ1 suppressed the cellular metabolism and blocked the production of cellular metabolic intermediates in B-ALL cells by downregulating the expression of c-MYC-mediated metabolic enzymes. Thus, these findings indicated that CDK7 was a potential clinical therapeutic target for B-ALL.

## Data Availability Statement

The datasets presented in this study can be found in online repositories. The names of the repository/repositories and accession number(s) can be found below: NCBI GEO GSE167099.

## Ethics Statement

The studies involving human participants were reviewed and approved by Ethics Committee of Shanghai Children's Medical Center. Written informed consent to participate in this study was provided by the participants' legal guardian/next of kin. The animal study was reviewed and approved by Animal ethics committee of Shanghai Children's Medical Center. Written informed consent was obtained from the owners for the participation of their animals in this study.

## Author Contributions

C-WD and KQ designed the study, analyzed and interpreted data, and wrote the paper. TA and M-HL performed experiments, analyzed and interpreted data. JX analyzed the RNA-SEQ and metabonomic data. HZ, W-WZ, NZ, R-YS, J-MZ, and L-TY performed experiments. LZ, LC, and KX discussed results and contributed to data interpretation. All authors read and approved the final manuscript.

## Conflict of Interest

The authors declare that the research was conducted in the absence of any commercial or financial relationships that could be construed as a potential conflict of interest.
