# Supplementary material for: CDK7 Inhibitor THZ1 Induces the Cell Apoptosis of B-Cell Acute Lymphocytic Leukemia by Perturbing Cellular Metabolism
Source: Front Oncol. 2021 Apr 6;11:663360. doi: 10.3389/fonc.2021.663360 (PMC8056175; doi:10.3389/fonc.2021.663360)
Supplement: Supplementary file 2 [file Table_1.DOCX]

Supplementary Material

CDK7 inhibitor THZ1 induces the cell apoptosis of B-cell acute lymphocytic leukemia by perturbing cellular metabolism

Tuersunayi Abudureheman, Jing Xia, Ming-Hao Li, Hang Zhou, Wei-Wei Zheng, Neng Zhou, Rong-Yi Shi, Jian-Min Zhu, Li-Ting Yang, Li Chen, Liang Zheng, Kai Xue, Kai Qing and Cai-Wen Duan

**Table S1**

Clinical data for primary B-ALLs

| Sample | Sample type | cytogenetics | Age at diagnosis | Gender | White cell count  (×10^9^ cells) |
| --- | --- | --- | --- | --- | --- |
| B-ALL1 | Primary | Fusion gene negative | 6 years | male | 43.2 |
| B-ALL2 | Primary | Fusion gene negative | 3 years | male | 58.5 |
| B-ALL3 | Primary | BCR/ABL | 10 years | famale | 126 |
